# On the Face of It: No Differential Sensitivity to Internal Facial Features in the Dog Brain

**DOI:** 10.3389/fnbeh.2020.00025

**Published:** 2020-03-03

**Authors:** Dóra Szabó, Anna Gábor, Márta Gácsi, Tamás Faragó, Enikő Kubinyi, Ádám Miklósi, Attila Andics

**Affiliations:** ^1^Department of Ethology, Eötvös Loránd Univesity, Budapest, Hungary; ^2^MTA-ELTE ‘Lendület' Neuroethology of Communication Research Group, Budapest, Hungary; ^3^MTA-ELTE Comparative Ethology Research Group, Budapest, Hungary

**Keywords:** dog, fMRI, visual, face-sensitive, face processing, inner face, DFA, face area

## Abstract

Dogs are looking at and gaining information from human faces in a variety of contexts. Next to behavioral studies investigating the topic, recent fMRI studies reported face sensitive brain areas in dogs' temporal cortex. However, these studies used whole heads as stimuli which contain both internal (eyes, nose, mouth) and external facial features (hair, chin, face-outline). Behavioral studies reported that (1) recognition of human faces by dogs requires visibility of head contour and that (2) dogs are less successful in recognizing their owners from 2D pictures than from real human heads. In contrast, face perception in humans heavily depends on internal features and generalizes to 2D images. Whether putative face sensitive regions in dogs have comparable properties to those of humans has not been tested so far. In two fMRI experiments, we investigated (1) the location of putative face sensitive areas presenting only internal features of a real human face vs. a mono-colored control surface and (2) whether these regions show higher activity toward live human faces and/or static images of those faces compared to scrambled face images, all with the same outline. In Study 1 (*n* = 13) we found strong activity for faces in multiple regions, including the previously described temporo-parietal and occipital regions when the control was a mono-colored, homogeneous surface. These differences disappeared in Study 2 (*n* = 11) when we compared faces to scrambled faces, controlling for low-level visual cues. Our results do not support the assumption that dogs rely on a specialized brain region for processing internal facial characteristics, which is in line with the behavioral findings regarding dogs inability to recognize human faces based on these features.

## 1. Introduction

While object perception is based on thedistributed response from neurons coding different aspects of an object, the neural response to faces in humans (Andrews et al., 2010) and in some other primate species (Burke and Sulikowski, 2013) seems to be somewhat exceptional. The holistic processing of faces is manifested at the behavioral level via three main phenomena. The inversion effect corresponds to impaired identification of upside-down faces compared to upright faces (Kanwisher et al., 1998). The part-whole effect implies better discrimination performance of two face parts when the parts are presented in the context of a whole face than when presented in isolation (Tsao and Livingstone, 2008). Finally, the composite effect, which means slower identification of half of a chimeric face if it is aligned with an inconsistent other half-face than if the two half-faces are misaligned (Tsao and Livingstone, 2008). These effects show that in the human brain, faces are represented not only as a combination of the parts (eyes, nose, mouth), but as a non-decomposable whole. These effects are disproportionately present for faces in contrast to e.g., everyday objects.

More than 20 years of research localized several brain areas in humans which play specific roles in face processing. Kanwisher et al. (1997) described the fusiform face area at the border of the temporal and occipital lobe as a region showing higher activations to faces than various non-face objects and scrambled images. Since then, researchers carried out a large number of studies to examine its characteristics. Based on the literature, the observed effect in face selective areas cannot be explained by differences in low-level spectral features (Andrews et al., 2010) or expert individuation (Rhodes et al., 2004). Despite the holistic representation of faces, this higher activation is more pronounced toward internal facial features (eyes, nose, mouth) when compared to external features (hair, chin, face-outline)(Kanwisher and Yovel, 2006; Andrews et al., 2010). Face-sensitive areas in humans are considered as being right lateralized in general (Kanwisher and Yovel, 2006), although a more recent study suggested that the response profiles of the left and right fusiform gyri differ, with the right fusiform gyrus being more involved in categorical face/non-face judgements, while the left fusiform gyrus reacting more to the level of similarity to faces in case of non-face images (face-semblance) (Meng et al., 2012).

Face processing has been also investigated in a wide range of non-human animals (mainly using faces of conspecifics as stimuli), both at the behavioral and neural level (Leopold and Rhodes, 2010; Burke and Sulikowski, 2013). Certain phenomena such, as the face inversion effect, were also observed in phylogenetically distant taxa (e.g., in a recent study on fish Kawasaka et al., 2019). However, the presence of a composite effect was reported only for primates (Burke and Sulikowski, 2013).

Family dogs are living in close day-to-day contact with humans, easily developing a cross-species, individualized communicative relationship with them. The properties of their visual communication (including initiating and maintaining eye contact) with humans is well-studied (Miklósi, 2009). Human facial cues are thought to be important in dog-human communication. Some recent behavioral studies showed dogs' ability to use human facial cues for inferring attentional states (Gácsi et al., 2004) and discriminate between emotional expressions (Nagasawa et al., 2011; Müller et al., 2015).

A study in dogs (Huber et al., 2013), where the authors trained dogs to discriminate between human heads based on visual information, reported that transitioning from live presentation to pictures of heads of the same individuals, and from these head pictures to pictures of faces showing only internal features of the head required additional training session before some of the subjects could master the new task.

Dilks et al. (2015) and Cuaya et al. (2016) compared portraits of humans to everyday objects in fMRI studies and reported higher activity toward faces in the right temporal regions (Dilks et al., 2015) or in bilateral temporal regions with greater extent in the left temporal lobe (Cuaya et al., 2016). Thompkins et al. (2018) compared dog and human portraits and reported separate brain areas in the left temporal cortex showing higher activity to portraits of humans and dogs, resepctively. Comparing heads with a wide variety of objects/scenes instead of a more restricted category (e.g., houses), may intoduce an unintended bias, as heads are more uniform in regard to their shape than the shape of different everyday objects. All of the above mentioned dog fMRI studies presented two-dimensional still images stimuli, but it has not been established whether or not dogs (whose visual capacities differ from that of humans) perceive basic two-dimensional stimuli in the same way as they do in case of three-dimensional stimuli (Byosiere et al., 2018).

In humans (who constantly look at images from a very early age), there is evidence that photos of objects are processed differently on the neural level (Snow et al., 2011), using real items amplifies value-based fMRI responses (Culham et al., 2013) and video-mediated faces do not elicit the physiological response (measured via skin conductance responses) detectable in case of live faces (Riby et al., 2012).

Presenting two-dimensional visual stimuli to animals is methodologically challenging (Leopold and Rhodes, 2010; Burke and Sulikowski, 2013). One of the concerns is whether in studies which rely on photographs, one can reasonably expect that animals see the image as a depiction of a real object without explicit training and/or a significant amount of previous experience with this type of stimuli (Burke and Sulikowski, 2013). The other concern is whether static stimuli has comparable ecological validity to dynamic stimuli in case of a carnivorous species.

**Figure 1:**
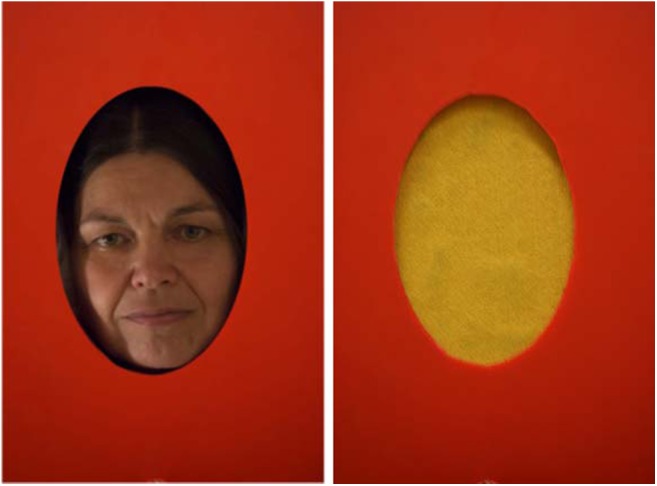
Stimuli used in Study 1. The individual shown on the figure is an author of this paper and consented to the publication of their photographs.

In the current study, our aim was to investigate face sensitivity in dogs via fMRI by using similar stimuli as in the study of Huber et al. (2013). Dogs were presented with features of the inner face (see Figure 1). To determine the level of similarity between face processing in dogs and humans, it is important to investigate what aspects of the stimuli are necessary to elicit a response in the analogous brain areas in dogs. The novelty of our design was that it allowed us to compare face and non-face stimuli, while all stimuli had the same size and shape. To make sure that dogs see portraits as a depiction of a real person/face (and to avoid explicit training and its potential impact), we decided to rely on live faces. First, as a proof of concept to test the viability of our setup and to locate candidate brain areas which respond with higher activity toward visually complex stimuli, we compared a live human face to a simple control with markedly different visual characteristics (Study 1, a mono-colored surface). Study 2 had two goals: (1) to test whether the regions showing higher activity toward a face in Study 1 also do so in case of a more stringent control (scrambled images), and (2) to compare the BOLD response when using a portrait (photo) and a live face as stimuli.

Our hypothesis was that, if the regions in the dogs' brain, which were previously reported as face-sensitive (Dilks et al., 2015; Cuaya et al., 2016; Thompkins et al., 2018), react to the configural information present in inner faces, this area would show higher activity toward faces even when we control for low level visual characteristics. Alternatively, if the involved brain areas react toward the variation in visual properties instead of face configuration/holistic face processing, these brain areas would not show higher activity for faces when compared to scrambled images with the same shape and color pattern.

## 2. Materials and Methods

### 2.1. Participants

In Study 1, we measured 13 family dogs *(Canisfamiliaris)* (age 5.31 ± 2.69 years (mean ± SD), range 1–11 years, 6 females and 7 males, 6 golden retrievers, 5 border collies, 1 Chinese crested dog, 1 Cairn terrier. Only subjects which already passed a run wise 3 mm motion threshold criteria during a 6-min-long functional data collection run for a previous study were enrolled. In addition to the MRI acoustic training, the dogs were prepared for the visual study design as follows: We gradually (with increasing durations) removed the visible human who had been sitting at the end of the scanner bore. The next step was that the human was eventually walking over in front of the dog lying in the scanner, to get the dogs used to a disappearing and reappearing human while they lied still in the scanner. Completing these steps took 2–6 sessions. We concluded their training when they were able to remain still for 6 min under these conditions. In Study 2, the same subjects participated, except 2 dogs (a 5 years old female Border collie and a 4 years old male golden retriever was not part of the second study). The training procedure was described in Andics et al. (2014), and it was based on individual and social learning using positive reinforcement.

### 2.2. Stimuli

**Figure 2:**
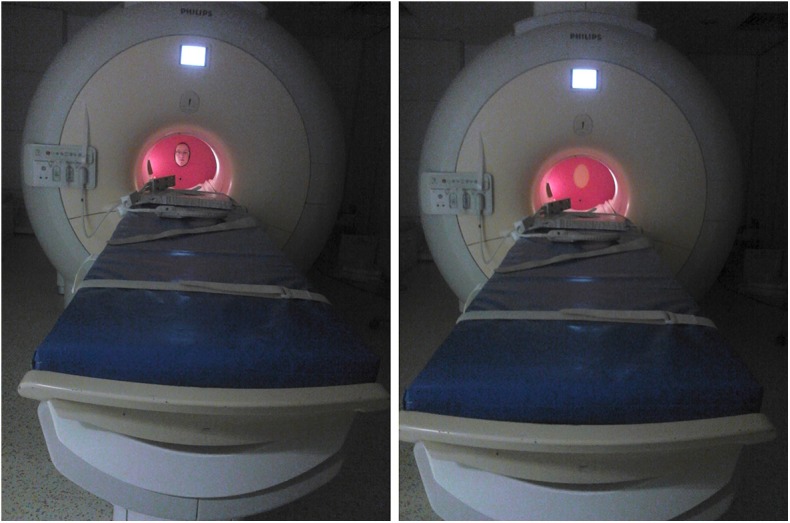
In-scanner setup of the equipment and stimulus presentation. The individual shown on the figure is an author of this paper and consented to the publication of their photographs.

**Figure 3:**
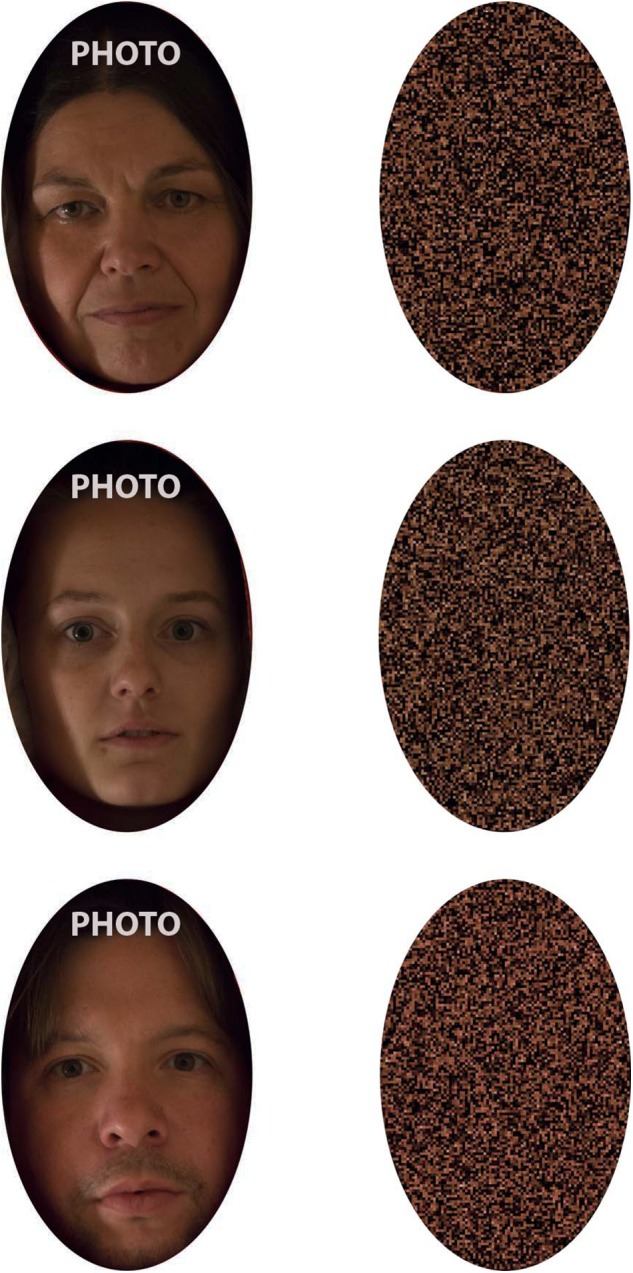
Stimuli used in Study 2. Each stimulus set contained a live presentation, a portrait from the same individual, and the scrambled stimuli generated from the portrait. The individuals shown on the figure are authors of this paper and consented to the publication of their photographs. A video demonstrating stimulus presentation in Study 2 is available at the following link:(https://www.youtube.com/watch?v=tNaKRsG72b0).

For Study 1, the stimuli consisted of a real-life face (the female trainer of the dogs) and a piece of yellow, mono-colored textured neoprene rubber sheet mounted on the stimulus presenting wheel (see Figure 2). For Study 2, we used three types of stimuli (real life faces, a pictures of the same faces and a scrambled images of the same faces, see Figure 3). A professional photographer took the pictures used in this study, in the actual study setup in the scanner to ensure that the conditions are as similar as possible. Stimulus set consisted of three separate series (1 male, 2 females, all Caucasian, all familiar to the dogs). In a single run, we used a single series (all three stimuli showing the same person) and every subject was tested with two different sets. The size and shape of all stimuli were the same: oval-shaped, with a height of 22.57 cm and width of 14.97 cm. Box scrambling was carried out in Matlab via randomly rearranging pixels with a pixel size of 1.5 by 1.5 mm. Video illustrations of stimulus presentation in Study 2 are available at the following links: (https://www.youtube.com/watch?v=tNaKRsG72b0)(view from the subject's side) (https://www.youtube.com/watch?v=DO1ELrupxmc)(view from the Experimenters' side).

In Study 1 (2 conditions), one run contained 50 stimulus presentations, while in Study 2 (3 conditions), one run contained 51 presentations. The stimuli were presented in a counterbalanced, semi-random order. Controlled presentation timing and duration were achieved via pre-recorded instructions which were played to the Experimenter (see below) through headphones during the runs. The instructed duration of stimulus presentation was 3,000 ms. The actual duration may have slightly varied across trials (see below). Inter-stimulus interval was jittered between 4,000–6,000 ms. When comparing our designs' detection power to the top 5 randomizations generated by optseq2 with these settings *post-hoc* (duration of run, range of ITI, number of stimuli) for the two studies combined, the detection power was nearly identical [optseq2 generated: 5.30 ± 0.31 (mean ± SD), our randomization: 5.31 ± 0.27 (mean ± SD)].

### 2.3. Procedure

We constructed a dismountable equipment for the purpose of this study. In Study 1, this included a plastic wall covering the MR scanner bore and a rotating plastic wheel for the presentation of the visual stimuli (a hole in case of a live face, see Figure 2). For Study 2, an additional lever was added to the equipment, which could be lifted to cover and uncover the stimuli. This assured that the presentations of stimuli started only after the person/stimuli reached a stationary position.

We utilized different wheels for Study 1 and Study 2, the former containing two stimulus presentation segments, while the latter containing three such segments. Stimulus presentation in Study 1 involved a single Experimenter, sitting at the end of the scanner bore, who served as the face stimulus and also operated the wheel based on pre-recorded instructions played via earphones. In Study 2, Experimenter 1 served as the stimulus just like in Study 1, while an additional Experimenter operated the equipment (both the lever and the wheel) based on the pre-recoded instructions played via earphones. Timing and instructions of the stimulus presentation were delivered binaurally through MRI-compatible sound-attenuating headphones (MR Confon, Magdeburg, Germany) and were controlled using Matlab (version 7.9) Psychophysics Toolbox 3. Dogs were provided with noise protection through MRI-compatible sound-attenuating headphones. All procedures were approved by the Government Office of Pest County Directorate of Food Chain Safety and Animal Health (XIV-I-001/520-4/2012), and conducted in accordance with the national and European guidelines for animal care. Owners of the pet dogs participated in the study on a voluntary basis with their dogs and gave their written informed consent to the MR scanning of their dogs.

### 2.4. Image Acquisition

MRI measurements were taken at the MR Research Centre of the Semmelweis University Budapest on a Philips Achieva 3 T whole body MR unit (Philips Medical Systems, Best, The Netherlands) with a Philips SENSE Flex Medium coil. This coil consisted of two elliptical elements that were 14 ×17 cm. One element was placed below the dog's head while the other element was fixed with plastic strips above the dog's head. In both experiments, we obtained EPI-BOLD fMRI time series via continuous scanning with the following parameters: TE = 36 msec, TR = 2,035 ms, FOV = 224 ×224 ×101 mm, Voxel size = 3.5 ×3.5 ×3.5 mm (including 0.5 mm slice gap). We collected 29 transverse slices in ascending order covering the whole brain. One run consisted of 180 scans and lasted for 360 s. For Study 1, we collected 1 run/subject, while Study 2 consisted of 2 runs.

### 2.5. Image Processing and Analysis

Image pre-processing and statistical analysis were performed using SPM12 (http://www.fil.ion.ucl.ac.uk/spm/). FMRI pre-processing and statistical analysis were performed using SPM12. Pre-processing included affine realignment (6 parameters, least square approach) and reslicing of the images of the individual runs, followed by manual coregistration of the mean image to the individuals' own structural T1 image in Amira 6.0 (Thermo Fisher Scientific). The individual structural images were normalized and transformed manually (linear, non-rigid transformation) to a custom-made individual template anatomical image (Czeibert et al., 2019) in Amira. The resliced images were then coregistered and normalized to this transformed mean functional image via SPM's standard nonlinear warping function with 16 iterations and smoothed with an FWHM of 5 mm.

Data were analyzed via a general linear model and statistical parametric mapping in SPM. Condition regressors were created for each run and for each condition. We modeled the trials as 3 s long events. We included realignment regressors for each run to model potential movement artifacts. We utilized a high-pass filter with a cycle-cutoff of 128 s to remove low-frequency signals. Regressors were convolved with the canonical haemodynamic response function in SPM. Single-subject fixed effect analyses were followed by group level, whole-volume random effects analyses. For the GLM, we calculated the average smoothness of the residuals using 3dFWHMx and then used 3dClustsim to determine the cluster threshold with. For Study 2, in case of higher-level contrasts, next to the whole-brain analysis, results where also evaluated in a restricted brain search space, including only the regions which higher activity for the face stimulus in Study 1, via an inclusive mask thresholded at *p* < 0.001. Lateralization indices (LI) were calculated using a bootstrapping method implemented in SPM's LI-Toolbox (Wilke and Lidzba, 2007). Subject-specific contrast images for each condition (compared to baseline) served as input, with an exclusive midline mask of 11 mm. The process resulted in an overall weighted bootstrapped LI per subject and per contrast. These LIs across subjects and conditions were then compared in one-sample *T*-tests to assess condition-specific hemispheric bias. To control for false positives, we performed family-wise error (FWE) correction for multiple comparisons using 3dFWHMx/3dClustSim modules of AFNI (http://afni.nimh.nih.gov/afni; “fixed” version, compiled Oct, 2019). Specifically, we run 10,000 Monte Carlo simulations to estimate the cluster size above which the false positive probability was below a given level (FWE = 0.05) for a given cluster defining threshold (*p* < 0.0001 in Study 1, *p* < 0.001, and *p* < 0.05 in Study 2, respectively).

## 3. Results

We only report clusters surviving family-wise error (FWE) correction for false-positives with alpha <0.05. The framewise displacement within our data was 0.21 ± 0.25 mm (mean ± SD). The distribution of framewise displacement values showed that the majority of the framewise displacement values fell well below 0.5 mm (the human standard for task-based fMRI studies). We censored runs if the total, cumulative motion during did exceed 3 mm in any direction. In this case we truncated the run, discarding scans from the end of the run. In Study 1, two runs were involved (after 120 and 160 scans out of 180, respectively), while in Study 2, a single run was involved (after 125 scans out of 180).

**Figure 4:**
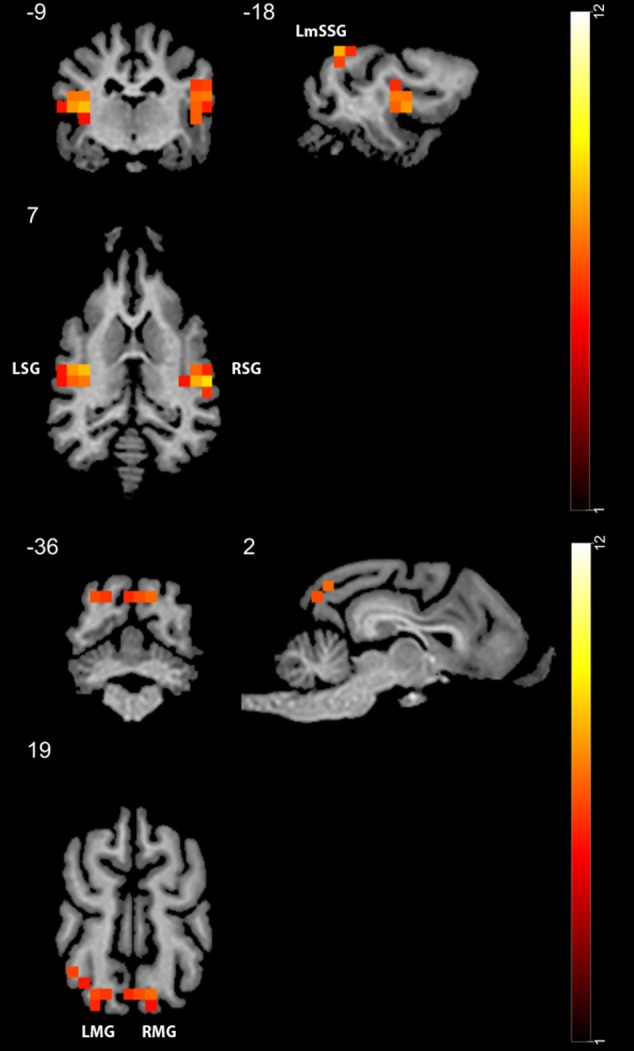
GLM results in study 1 (*n* = 13). Regions showing higher activity to the face stimulus compared to the mono-colored control. Color heatmap and color bar indicates *t*-values, displayed on selected slices overlaid on the template brain. Thresholdet at uncorrected voxel threshold *p* < 0.0001, FWE corrected at the cluster level. RSG, right Sylvian gyrus; LmSSG, left mid suprasylvian gyrus; LMG, left marginal gyrus; RMG, right marginal gyrus; LSG, left Sylvian gyrus.

In Study 1, we found higher activity for the face condition compared to the control condition (a mono-colored surface) in multiple temporal and occipital regions (see Figure 4 and Table 1, results reported with an uncorrected peak-level *p* < 0.0001, clusters of min. 2 voxels), while we found no supra-threshold regions which would have shown higher activity to the control with the same threshold. The regions showing higher activity toward the face condition included the bilateral caudal and rostral regions of the Sylvian gyrus, the bilateral marginal gyrus, the left mid-suprasylvian gyrus and the insular cortex. The caudal part of the marginal gyrus is involved specifically in the visual processing, as containing the primary visual area, the insular cortex is a primary viscerosensory area receiving inputs from the internal organs, whilst the Slyvian gyrus and the middle suprasylvian gyrus are association areas next to the primary somatosensory and auditory cortexes, thus having the capability to integrate stimuli from different sensory modalities.

**Table 1:** Main GLM results for Study 1 with a threshold of peak-level uncorrected *p* < 0.0001, showing the results of the face > controll contrast.

**Brain region**	**cluster-level p** **(FWE-corrected)**	**Cluster size (voxels)**	**Peak T**	**Peak-level p (FWE-corrected)**	**Coordinates (x, y, z)**
R rostral Sylvian gyrus, caudal Sylvian gyrus	<0.001	28	8.74	0.002	23, −12, 6
Insular cortex, L rostral Sylvian gyrus, caudal Sylvian gyrus	<0.001	14	8.65	0.002	−16, −12, 9
L mid suprasylvian gyrus	0.003	7	7.91	0.005	−19, −29, 23
L marginal gyrus			6.27	0.046	−12, −36, 20
R marginal gyrus	0.003	7	7.32	0.010	6, −33, 23

*We found no significant cluster in case of control > face contrast. One cluster may contain multiple peaks. In this case, providing cluster sizes for the peaks separately is not possible. L mid suprasylvian gyrus and L marginal gyrus belong to the same cluster, hence we could not report the cluster sizes separately for these two peaks*.

However, in Study 2 we found no supra-threshold voxels with a threshold of *p* < 0.001 in any of the investigated contrasts. No regions showed higher activity toward a face when compared to a more stringent control (scrambled images), and no differences emerged between the portrait (photo) and the live face presentations. This was also the case when we restricted brain search space to the inclusive mask based on the results of Study 1. With a weaker threshold of p(uncorrected) <0.05, still no regions in any of the investigated contrasts showed higher activity toward faces (neither the live nor the portrait face conditions). A cluster in the left caudate nucleus showed higher activity toward scrambled images in both the combined faces vs. scrambled images (coordinates −2, 6, 6, size: 62 voxels) and in the portraits vs. scrambled images contrast (coordinates −2, 6, 6, size: 45 voxels). In the portraits vs. scrambled images contrast, an other cluster located in the right sensorymotoric cortex also showed higher activity toward scrambled images (coordinates 13, 1, −20, size: 36 voxels). The boot-strapping approach revealed no group level lateralization of cortical responses in any of the conditions within the two experiments (*ps* > 0.1).

## 4. Discussion

To our knowledge, this is the first study which investigated human face processing in dogs via fMRI using exclusively inner face stimuli. In Study 1 we found multiple, bilateral brain regions in the temporal and occipital cortex which showed higher activity toward the inner parts of a single face than to a mono-colored control surface. This is in line with previous human results, reporting higher activity toward faces than surfaces/textures in humans, showing that even contrasts with such low level controls activate these higher-level regions in the human fusiform face areas (Puce et al., 1996). In contrast, we found no such effect in Study 2 when the stimuli contained images from multiple individuals and we also controlled for color and brightness. This was also the case when we restricted our analysis to the regions of interests based on the findings of the first study. The temporal regions displaying higher activity toward faces in Study 1 did not show higher activity toward faces when compared to the scrambled images in Study 2. We found no differences in activity between the live faces and portraits conditions. Live presentation might have shown small deviations from the static portrait (even though the human demonstrator always presented a neutral facial expression). We addressed this issue by including an “intermediate” portrait condition which is (1) arguably highly similar visually to the live condition and (2) appropriately controlled by the scrambled face. Note that we did not find significant differences between brain activities for processing live faces and portraits (both conditions containing faces, but one containing more variability regarding the stimuli) and neither of them differed from the scrambled condition in our study. While it is theoretically possible that the minimum cluster size determined by 3dFWHMx and 3dClustsim (2 voxels in case of Study 1) represent an area larger than dogs face processing area, the previous studies reported significant activation toward faces in areas larger than this, using similar voxel sizes and FWHMs (Dilks et al., 2015; Cuaya et al., 2016).

Both the temporal and occipital regions detected in Study 1 showed similar localization to those reported by previous fMRI studies investigating face sensitive areas in dogs (Dilks et al., 2015; Cuaya et al., 2016; Thompkins et al., 2018), confirming that we measured a similar phenomenon with our setup. However, Study 2 showed that these regions do not show significantly higher activity toward faces when we control for low level visual characteristics via scrambling. This finding is in line with the results of Dilks et al. (2015), who also did not find significant difference in dogs' brain activity between images of faces vs. scrambled faces. In a behavior study by Pitteri et al. (2014), where the authors had trained dogs for discriminating the picture of their owner, they found that dogs experienced in this type of task were also able to pick their owners image from scrambled images, while naive dogs could not. This suggests that when presented with a similar visual discrimination training situation, dogs may learn to master such tasks relying on other local visual processes not specific to face processing.

Although Pitteri et al. (2014) trained dogs to discriminate isolated face parts, a comparison between their performance related to isolated face parts vs. whole faces is not possible, as every subject was trained/tested with whole heads only after they mastered all three isolated face part discrimination tasks, which means that the presence of a part-whole effect could not be tested. Currently, there are no publications showing disproportionate inversion effect for faces (Racca et al., 2010 reported a non-specific-inversion effect; dogs showed the same viewing pattern to inverted objects than to human faces), part-whole effect or composite effect in dogs, which would signal configural/holistic processing of faces (Burke and Sulikowski, 2013).

Similarly to Cuaya et al. (2016), but unlike Thompkins et al. (2018) we could not record the dogs' in-scanner viewing behavior to evaluate the spatial viewing pattern of our participants (although the presentation of live stimuli allowed us to have some feedback regarding the dogs' attentional state and they seemed to be attentive). In Study 2, we used a more diverse stimulus set, multiple runs, more conditions and a smaller sample size than in Study 1, which may have affected statistical power. Our study differed in several aspects from previous designs. We utilized an event-related design, while Dilks et al. (2015) and Cuaya et al. (2016) utilized a block design. While previous studies had a systematic difference between face and non-face objects regarding shape and ratio of real-size to projected size (more variability in shape and other visual characteristics among objects, faces are in general projected to be approximately real sized while objects not), we controlled for this effect in our study, which may in part explain the different findings. While our sample size may be considered low compared to the standards of human studies, it exceeds the sample size utilized in previous similar studies and considerably larger than several primate study's. We included dogs varying in breeds, sex and age: this includes sample variability and therefore increases generalizability of our findings, but it potentially also leads to larger individual variability, perhaps reducing power.

We found no signs of lateralized brain responses in either study. Previous dog face fMRI studies reported varying effects regarding face sensitive areas: only in the left hemisphere (Thompkins et al., 2018), only in the right hemisphere (Dilks et al., 2015) or bilateral activation with greater extent in the left lobe (Cuaya et al., 2016).

Dogs are looking at and gaining information from human faces in a variety of contexts (Adachi et al., 2007; Huber et al., 2013), and they are able to rely on cues provided by a (life-size) projected human (Pongrácz et al., 2003). We do not debate that dogs are able to process and extract relevant information from (projected) faces, but based on our results we claim that the current evidence is not sufficient to argue that dogs utilize holistic face processing with a specialized brain region showing higher activity selectively to faces. Alternatively, family dogs without explicit, specialized training may rely on other characteristics (head shape), context (dogs in general have limited experience with isolated heads without a body) or combined information from different modalities to detect relevant social cues. This is also supported by a behavioral study showing that dogs are only able to spontaneously discriminate between their owner's and a stranger's head if outer face elements are also visible (Mongillo et al., 2017). A distributed response from neurons coding different aspects of the stimuli is proved to be sufficient in a variety of contexts in humans from solving an individuation task of Lepidoptera images (Rhodes et al., 2004) to processing Chinese characters (Fu et al., 2012), showing that a highly specialized, brain level adaptation is not a prerequisite for compelling cognitive performance. In a similar manner, dogs' expertise when engaging in social interactions with humans do not necessary require the presence of the same highly specialized brain adaptations found in humans.

## Data Availability Statement

The datasets ANALYZED for this study can be found in the OSF repository (https://osf.io/jvct4/?view_only=7a665871ce8b4181afe364bec78f5f3d).

## Ethics Statement

This study was carried out in accordance with the recommendations of the national and European guidelines for animal care. The protocol was approved by the Government Office of Pest County Directorate of Food Chain Safety and Animal Health (XIV-I-001/520-4/2012).

## Author Contributions

DS: conceptualization, methodology, formal analysis, investigation, writing original draft, writing review and editing, project administration. AG: methodology, writing review and editing. MG: conceptualization, investigation, writing review and editing, visualization. TF: methodology, writing review and editing. EK: writing review and editing, supervision, and funding acquisition. ÁM: writing review and editing, supervision, and funding acquisition. AA: conceptualization, methodology, formal analysis, investigation, and supervision.

### Conflict of Interest

The authors declare that the research was conducted in the absence of any commercial or financial relationships that could be construed as a potential conflict of interest.
